# Empagliflozin Mitigates Doxorubicin-Induced Cardiotoxicity in Rats: Electrocardiographic, Biochemical, and Histopathological Evidence

**DOI:** 10.3390/ijms27073090

**Published:** 2026-03-28

**Authors:** Iacob-Daniel Goje, Valentin Laurențiu Ordodi, Greta-Ionela Goje, Florina Maria Bojin, Andrei-Dragoș Crăciun, Daniela Crîsnic, Mihnea Derban, Andreea Severina Barbulescu, Valentina Gabriela Ciobotaru, Virgil Păunescu, Daniel-Florin Lighezan

**Affiliations:** 1 Department of Medical Semiology I, “Victor Babeș” University of Medicine and Pharmacy, No. 2 Eftimie Murgu Square, 300041 Timisoara, Romania; daniel.goje@umft.ro (I.-D.G.); vaciobotaru@gmail.com (V.G.C.); dlighezan@umft.ro (D.-F.L.); 2 Advanced Cardiology and Hemostaseology Research Center, “Victor Babeș” University of Medicine and Pharmacy, No. 2 Eftimie Murgu Square, 300041 Timisoara, Romania; barbulescu.greta@umft.ro; 3 Immuno-Physiology and Biotechnologies Center, Department of Functional Sciences, “Victor Babeș” University of Medicine and Pharmacy, No. 2 Eftimie Murgu Square, 300041 Timisoara, Romania; florinabojin@umft.ro (F.M.B.); crisnic.daniela@umft.ro (D.C.); vpaunescu@umft.ro (V.P.); 4 Faculty of Industrial Chemistry and Environmental Engineering, “Politehnica” University Timisoara, No. 2 Victoriei Square, 300006 Timisoara, Romania; 5 OncoGen—Center for Gene and Cell Therapies in Cancer Treatment, Clinical Emergency County Hospital “Pius Brinzeu” Timisoara, No. 156, Liviu Rebreanu, 300723 Timisoara, Romania; andrei.craciun@student.umft.ro; 6 Department I of Nursing, University Clinic of Clinical Skills, “Victor Babeș” University of Medicine and Pharmacy, No. 2 Eftimie Murgu Square, 300041 Timisoara, Romania; 7 Faculty of General Medicine, “Victor Babeș” University of Medicine and Pharmacy, No. 2 Eftimie Murgu Square, 300041 Timisoara, Romania; 8 Department of Pathology, CF Clinical Hospital, 300310 Timisoara, Romania; mihneaderban@gmail.com; 9 Center for Advanced Research in Gastroenterology and Hepatology, “Victor Babeș” University of Medicine and Pharmacy, 300041 Timisoara, Romania; barbulescu.andra91@gmail.com

**Keywords:** cardio-oncology, cardiotoxicity, SGLT2 inhibitors, anthracyclines, doxorubicin

## Abstract

Doxorubicin (DOX) is a widely used anthracycline, but its clinical use is limited by dose-dependent cardiotoxicity. This experimental study evaluated the cardioprotective potential of empagliflozin (EMPA) against DOX-induced cardiotoxicity. Thirty healthy adult rats were randomized into five groups (*n* = 6): control (group I), EMPA (group II), EMPA + DOX (group III), DOX (group IV), and EMPA-preconditioning + DOX (group V). EMPA was administered orally at 10 mg/kg/day, either concomitantly with DOX or as a 14-day preconditioning course. Cumulative DOX exposure reached 15 mg/kg to establish a reproducible cardiotoxicity model. Serial electrocardiograms (ECGs) were recorded, blood samples were collected, and hearts were harvested for detailed histopathological analysis. Compared with the control group, group IV demonstrated significant QT/QTc prolongation and repolarization abnormalities, marked troponin elevation, and characteristic histological lesions, including cardiomyocyte vacuolization, loss of striations, diffuse inflammation, myocyte atrophy, and increased fibrosis. In groups receiving EMPA with DOX exposure (groups III and V), ECG changes were attenuated, troponin elevation was lower, and structural myocardial damage was substantially reduced, with better preservation of cardiomyocyte architecture and less fibrosis. These results suggest that EMPA provides significant cardioprotection against DOX-induced cardiotoxicity in rats, supporting further investigation of SGLT2 inhibitors in cardio-oncology.

## 1. Introduction

Heart failure (HF) linked to cancer treatment has become one of the leading causes of non-cancer-related morbidity and mortality as overall survival improves. Therapy with anthracyclines and other cardiotoxic cytostatics often limits the intensity of cancer treatment, underscoring the growing interest in pharmacological strategies for cardioprotection [1,2].

In recent years, the literature has highlighted a significant increase in preclinical and clinical studies exploring cardioprotective therapies in cancer patients treated with anthracyclines, particularly doxorubicin (DOX) [3]. DOX remains an essential agent in many cancer treatment regimens. Still, its side effect of inducing cardiomyopathy, arrhythmias, and HF through mechanisms involving oxidative stress, mitochondrial damage, and cardiomyocyte apoptosis is well documented [4,5]. Anthracyclines also cause a strong inflammatory response in the heart muscle. This involves activating pattern-recognition pathways, such as TLR2, TLR4, and the NLRP3 inflammasome. As a result, pro-inflammatory cytokines such as TNF-α, IL-1β, and IL-6 are released. These cytokines increase oxidative stress, cell death, and fibrotic changes, which, together, worsen ventricular function [6].

Sodium-glucose cotransporter 2 (SGLT2) inhibitors were initially developed for the treatment of type 2 diabetes mellitus (T2DM) by reducing renal glucose reabsorption [7]. Subsequently, clinical studies demonstrated substantial cardiovascular benefits, independent of glycemic status, leading to the approval of these agents in all major phenotypes of HF [8]. This profile has fueled the hypothesis that SGLT2 inhibitors may also play a protective role in chemotherapy-induced cardiotoxicity [9].

The proposed mechanisms of cardioprotection include optimizing cardiac metabolism, reducing oxidative stress, limiting myocardial inflammation, protecting against fibrosis and ventricular remodeling, and maintaining endothelial function [10]. Through this multimodal action, SGLT2 inhibitors may represent an attractive therapeutic option in the prevention and mitigation of cancer therapy-related cardiac dysfunction (CTRCD), especially when reducing or stopping chemotherapy is not feasible. Compared to classic strategies (beta-blockers, statins, renin-angiotensin system inhibitors), SGLT2 inhibitors are emerging as a modern alternative with good tolerability and potential for integration into the cardio-oncological arsenal [11,12].

Empagliflozin (EMPA), a SGLT2 inhibitor with favorable hypoglycemic and cardiovascular effects, has demonstrated consistent benefits on prognosis in patients with chronic HF, with or without T2DM, when added to guideline-directed medical therapy (GDMT). In the EMPEROR-Reduced and EMPEROR-Preserved studies, EMPA significantly reduced the composite risk of cardiovascular death and hospitalization in both HF with reduced ejection fraction (HFrEF) and HF with preserved ejection fraction (HFpEF), with a substantial reduction in recurrent hospitalizations [13,14]. Similarly, in EMPA-REG OUTCOME, EMPA reduced major cardiovascular events, including cardiovascular mortality and HF hospitalizations, reinforcing its cardioprotective profile [15]. Following these clinical observations, several experimental and mechanistic studies have begun to explain how EMPA might protect the heart at the cellular level, beyond simply lowering blood sugar. DOX-induced cardiotoxicity is tightly linked to suppression of the Nrf2 antioxidant pathway, leading to exacerbated oxidative stress, impaired autophagy, and increased cardiomyocyte death in experimental models. EMPA has been shown in diabetic cardiomyopathy models to enhance Nrf2 signaling and upregulate downstream antioxidant targets, thereby attenuating oxidative stress and mitochondrial dysfunction in cardiac tissue [16]. Very recent preclinical data also suggest that EMPA can alleviate DOX-induced cardiotoxicity by modulating JNK/Nrf2 signaling and reducing ROS burden [17]. Another intracellular signaling pathway of particular interest in this context is Adenosine monophosphate-activated protein kinase (AMPK), whose downregulation by DOX has been linked to impaired mitochondrial biogenesis, defective autophagy, and enhanced cardiomyocyte apoptosis. In a rat model of DOX cardiotoxicity, EMPA improved systolic ventricular function, reduced serum injury markers, and attenuated oxidative stress while activating the AMPK/SIRT-1/PGC-1α signaling axis in myocardial tissue, consistent with restoration of mitochondrial biogenesis and energy metabolism [18]. This data suggests that AMPK-dependent pathways represent a key node through which EMPA may oppose the energetic collapse and mitochondrial damage induced by DOX [19]. Inflammasome-mediated inflammation, particularly via nucleotide-binding domain-like receptor protein 3 (NLRP3), has emerged as an additional contributor to DOX-induced cardiotoxicity. Experimental HF models have demonstrated that EMPA reduces NLRP3 inflammasome activation and associated markers of sterile myocardial inflammation, with parallel improvements in LVEF, even in the absence of diabetes [20].

The validation of the cardioprotective effects has been conducted in vivo and in vitro research, which, since 2019, has highlighted the ability of EMPA to limit myocardial damage induced by DOX, known for its marked cardiotoxic profile and widespread use, in various experimental models—from acute and chronic toxicity in non-diabetic animal models [21,22,23,24].

In the present study, we aimed to evaluate the protective effects of EMPA against DOX-induced cardiotoxicity using electrocardiographic, biochemical, and histological parameters in non-diabetic Sprague-Dawley rats. The design of this animal study provides a solid framework for assessing DOX-induced cardiotoxicity and the cardioprotective potential of EMPA. The distribution of animals into five groups allows direct comparisons of anthracycline exposure across different EMPA cardioprotective strategies. Our protocol includes a pre-conditioning arm (group V) in which EMPA is initiated 14 days before DOX and continued during exposure, allowing a direct comparison between concomitant versus pre-treatment + concomitant strategy, an aspect not systematically addressed in previous EMPA/DOX studies and directly relevant to the timing of cardioprotective interventions in the clinical setting. Serial ECG monitoring, with detailed analysis of the corrected QT interval and repolarization segments, revealed repolarization abnormalities in the DOX-group and significant improvement in the groups receiving combined EMPA, suggesting a protective effect on myocardial electrical stability. These functional data were correlated with the dynamics of serum biomarkers, in particular, increased troponin levels in the DOX group and lower values when exposed to EMPA. Histopathological analysis reinforced these observations, showing cardiomyocyte vacuolization and loss of striation, diffuse inflammation, cardiomyocyte atrophy, and more extensive fibrosis in the DOX group, with markedly attenuated injury in the presence of the SGLT2 inhibitor. Through this multimodal approach, the study provides strong arguments that empagliflozin can modulate both acute and chronic doxorubicin-induced remodeling, opening real prospects for its prophylactic use in cardio-oncology.

At this point, the available evidence does not yet support including SGLT2 inhibitors as a standard strategy for cardio-oncological prophylaxis in guidelines. However, the current results provide a solid foundation for the efficacy of SGLT2 inhibitors in cancer patients, particularly in combination with anthracyclines.

## 2. Results

### 2.1. Animal Distribution and Baseline Data

**Figure 1:**
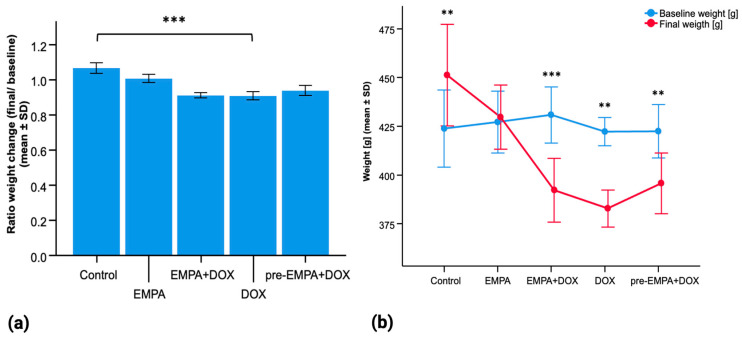
Bodyweight changes in the five groups. Control (Group I, normal saline; 14-day protocol), EMPA (Group II, 10 mg/kg empagliflozin; 14-day protocol), EMPA + DOX (Group III, 10 mg/kg empagliflozin + 15 mg/kg doxorubicin cumulative dose; 14-day protocol), DOX (Group IV, 15 mg/kg doxorubicin cumulative dose; 14-day protocol), pre-EMPA + DOX (Group V, preconditioning phase with daily EMPA for 14 days, followed by the EMPA/DOX protocol; 28-day protocol). (**a**) Independent sample *t*-test ratio weight change. DOX vs. Control (*p* < 0.001); DOX vs. EMPA + DOX (*p* = 0.786); DOX vs. pre-EMPA + DOX (*p* = 0.117); EMPA + DOX vs. pre-EMPA + DOX (*p* = 0.091). (**b**) Paired *t*-test per group baseline weight compared with final weight. Significant weight increase in Control (*p* = 0.004); significant weight decrease in EMPA + DOX (*p* < 0.001), DOX (*p* = 0.004), and pre-EMPA + DOX (*p* = 0.003). ** *p* < 0.01, and *** *p* < 0.001 compared with the indicated groups.

A total of 30 adult Sprague Dawley rats were included in the study and assigned to five experimental groups: group I (Control), group II (Empagliflozin), group III (Empagliflozin + Doxorubicin), group IV (Doxorubicin), and group V (Empagliflozin Preconditioning + Doxorubicin) (Figure 1).

Analysis of body weights showed that at the start of the experiment (day 1), there were no statistically significant differences between groups, indicating similar baseline characteristics. The protocol was completed on day 14 for the first four experimental groups and on day 28 for group V.

During the experimental protocol, three deaths were recorded: two from the DOX group (IV) and one from the EMPA + DOX group (III). No dedicated necropsy was performed at the time of death. Based on group allocation and the associated clinical/toxicity context, these deaths were considered treatment-related to doxorubicin.

Rats were expected to gain weight over time under normal growth conditions, as final body weight was assessed 14 days after baseline in groups I–IV and 28 days after baseline in group V. Consistent with this, control animals showed increase in body weight (423.8 ± 19.85 g vs. 451.2 ± 26 g, *p* = 0.004), whereas EMPA-treated rats had stable weight (427.16 ± 15.8 g vs. 429.6 ± 16.4 g, *p* = 0.82). In contrast, the EMPA + DOX group (group III) exhibited a significant reduction in body weight compared with baseline (432.0 ± 13.16 g vs. 392.2 ± 16.3 g, *p* < 0.001); the pre-EMPA + DOX group (group V) showed a trend towards weight loss over the longer 28-day period (422.4 ± 13.7 g vs. 395.7 ± 15.6 g, *p* = 0.003). The DOX group (group III) also showed a decrease in weight loss (423.4 ± 6.1 g vs. 382.85 ± 9.48 g, *p* = 0.004), consistent with DOX-induced cachectic effects.

The change in body weight after treatment for each rat group can be more easily appreciated in Figure 1, which graphically depicts the evolution of mean body weight over the entire experimental protocol (Figure 1).

### 2.2. EMPA Improves Electrocardiographic Parameters in DOX-Treated Rats

**Figure 2:**
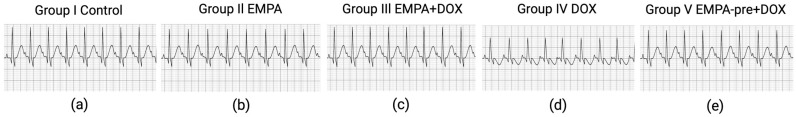
Electrocardiographic comparisons of all groups: (**a**) Control group: QRS segment, PR, QT, and QTc intervals, and R and T amplitudes were within the normal range. (**b**) The EMPA group was comparable to the control group. (**c**) In the EMPA + DOX group, prolongation of QRS, PR, and QTc intervals was significantly prevented, as well as amplitude modification of R and T waves in comparison with the DOX group. (**d**) In the DOX group, changes in all ECG parameters were observed, including decreases in HR and widening of the QRS, PR, QT, and QTc intervals, as well as changes specific to doxorubicin-induced cardiotoxicity affecting the amplitude of the R and T waves. (**e**) In the EMPA-pre + DOX group, prolongation of QRS, PR, QT, and QTc intervals was significantly prevented, as well as amplitude modification of R and T waves in comparison with the DOX group. Paper speed = 60 mm/s; paper voltage (amplitude) calibration = 10 mm/mV.

In this experimental model, electrocardiographic assessment was conducted in all five rat groups. The analysis identified ECG parameters indicative of DOX-induced cardiotoxicity, including conduction and repolarization abnormalities (Figure 2).

Electrocardiographic analysis revealed significant alterations in doxorubicin-treated animals compared with the control. In group IV, heart rate was significantly reduced (276.8 ± 10.4 bpm vs. 301.0 ± 6.2 bpm in control; *p* = 0.0111). At the same time, marked prolongation of the QRS segment (29.65 ± 1.80 ms vs. 19.98 ± 1.10 ms; *p* = 0.0004), PR interval (57.80 ± 2.91 ms vs. 42.15 ± 1.68 ms; *p* = 0.0004), and QT interval (125.10 ± 7.89 ms vs. 74.98 ± 2.50 ms; *p* = 0.0006) could be observed. The corrected QT interval (QTc) was substantially prolonged (268.7 ± 19.4 ms vs. 167.9 ± 4.7 ms; *p* = 0.0014). Additionally, R-wave amplitude was significantly decreased in DOX-treated animals (0.57 ± 0.03 mV vs. 0.87 ± 0.06 mV; *p* < 0.0001), and notably, T-wave amplitude became negative (−0.19 ± 0.03 mV vs. 0.34 ± 0.01 mV; *p* = 0.0013), indicating repolarization abnormalities (Table 1).

Empagliflozin treatment substantially attenuated these doxorubicin-induced electrocardiographic alterations. In group V, QRS duration was reduced (22.37 ± 1.05 ms; *p* = 0.0013 vs. group IV), PR interval (45.50 ± 1.30 ms; *p* = 0.0017 vs. group IV), and QT interval was shortened (85.84 ± 3.39 ms; *p* = 0.0010 vs. group IV). QTc interval was markedly improved (188.1 ± 6.5 ms; *p* = 0.0024 vs. group IV). Most notably, R-wave amplitude was preserved (0.82 ± 0.06 mV; *p* < 0.0001 vs. group IV), approaching normal values, and T-wave amplitude remained positive (0.34 ± 0.03 mV; *p* = 0.0002 vs. group IV).

Similarly, in group III, empagliflozin provided significant protection, with QRS (*p* = 0.0007), PR (*p* = 0.0006), QT (*p* = 0.0007), QTc interval (*p* = 0.0020), R-wave (*p* < 0.0001), and T-wave amplitude (*p* = 0.0002) all showing significant improvements compared to group IV (Table 1).

**Figure 3:**
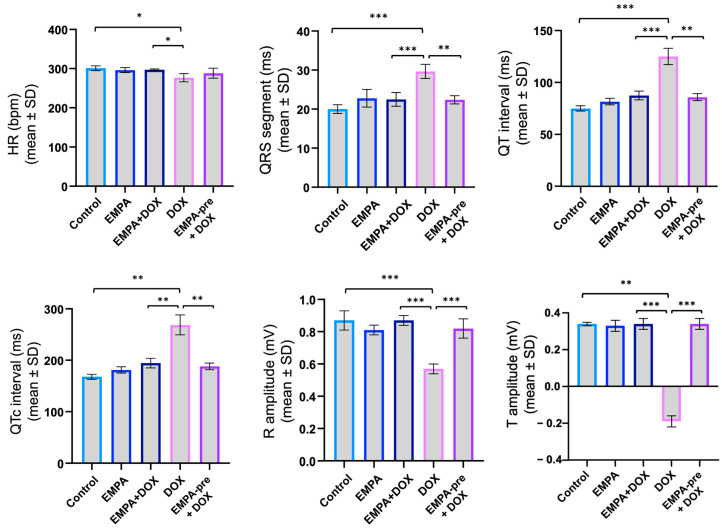
Electrocardiographic parameters in the indicated groups of rats. * *p* < 0.05, ** *p* < 0.01, and *** *p* < 0.001 compared with the indicated groups. bpm = beats per minute; HR = heart rate; ms = milliseconds; mV = millivolt; SD = standard deviation.

The electrocardiographic changes observed across the experimental groups highlight both the cardiotoxic effects of DOX and the cardioprotective effects of EMPA. Comparing group IV (DOX) with group I (control) highlights depolarization and repolarization abnormalities, whereas comparing group IV with the empagliflozin-treated groups shows a reduction in these changes, consistent with a cardioprotective profile. There were no statistically significant differences between groups III and V, indicating that EMPA exerted cardioprotective effects in both settings, but did not demonstrate a clear benefit of preconditioning in terms of electrocardiographic parameters. The graphical representation of the results not only allows the visualization of overall trends but also clearly highlights statistically significant differences between groups, facilitating the integrated interpretation of ECG data in the context of the anthracycline-induced cardiotoxicity model (Figure 3).

### 2.3. EMPA Reduces Troponin Levels in DOX-Induced Cardiotoxicity

Administration of doxorubicin as monotherapy caused leukopenia, with a decrease in white blood cell count from 6.21 ± 2.27 × 10^9^/L in the control group to 0.57 ± 0.23 × 10^9^/L in group IV (*p* = 0.002), suggesting marked myelosuppression in the white blood cell line. Severe anemia and thrombocytopenia were observed, reflected by reductions in red blood cells (RBC) (4.86 vs. 8.35 × 10^12^/L), hemoglobin (HGB) (8.20 vs. 14.40 g/dL), and platelets (PLT) (35.0 vs. 961 × 10^9^/L), all with *p* < 0.001. These changes indicate an extensive toxic effect of doxorubicin on the hematopoietic marrow. AST is elevated in the DOX group, compared to the control (*p* = 0.004). Although AST was significantly increased in the DOX group, ALT and LDH did not show the same pattern, suggesting that AST predominantly reflects myocardial and muscle damage. Troponin I is significantly higher in animals treated with DOX (0.20 ± 0.03 vs. 0.09 ± 0.02 ng/mL; *p* = 0.002), confirming acute myocardial injury consistent with anthracycline cardiotoxicity (Table 2).

The addition of empagliflozin (Group III) did not deliver significant changes in hematological parameters, indicating that it does not mitigate doxorubicin-induced myelosuppression. Also, AST, ALT, and LDH levels were comparable between the two groups (group III vs. group IV). In contrast, troponin I was significantly lower in the EMPA + DOX group than in the DOX monotherapy group (0.14 ± 0.04 vs. 0.20 ± 0.03 ng/mL; *p* = 0.037), supporting a cardioprotective effect of empagliflozin (Table 2).

In the empagliflozin preconditioning group, pretreatment does not prevent myelosuppression. The enzyme profile (AST, ALT, LDH) is also comparable between groups IV and V (*p* > 0.7), with no clear evidence of liver protection. Troponin I concentrations are significantly lower in Group V than in Group IV (0.13 ± 0.02 vs. 0.20 ± 0.03 ng/mL; *p* = 0.010). This suggests that preconditioning with empagliflozin confers a robust cardioprotective effect, reducing myocardial injury (Table 2).

There were no statistically significant differences in troponin dynamics between groups III and V, indicating that EMPA reduced DOX-induced myocardial injury in both regimens, but without providing evidence that preconditioning leads to better troponin reduction (*p* = 0.6303).

**Figure 4:**
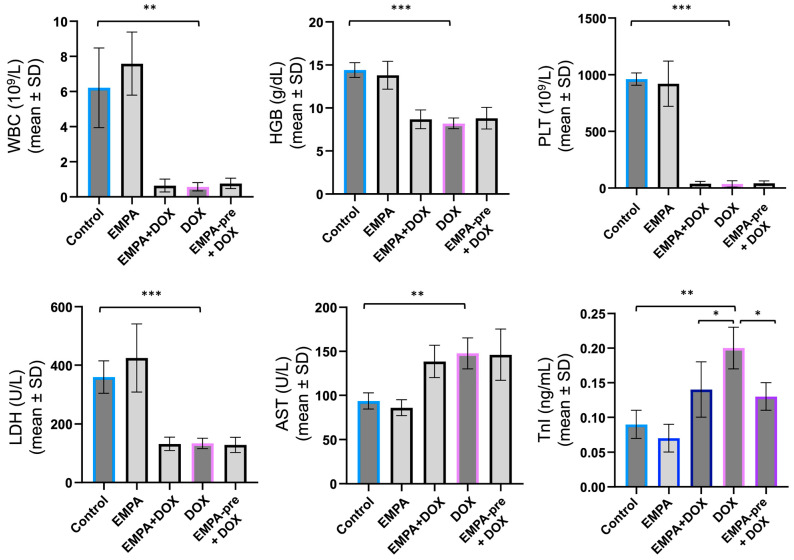
Blood parameters in the indicated groups of rats. * *p* < 0.05, ** *p* < 0.01, and *** *p* < 0.001 compared with the indicated groups. AST = aspartate aminotransferase; HGB = hemoglobin; LDH = lactate dehydrogenase; PLT = platelets; SD = standard deviation; TnI = troponin I; WBC = white blood cells.

Graphical illustration of blood parameters, especially troponin, provides a much more precise understanding of myocardial injury dynamics across groups. The troponin rise in group IV (DOX) compared to the control group highlights the cardiotoxic profile of anthracyclines, while the attenuation of this trend in the empagliflozin-treated groups supports the cardioprotective effect of the SGLT2 inhibitor (Figure 4).

### 2.4. EMPA Mitigates Acute and Chronic DOX-Induced Histopathological Myocardial Injury

To investigate the functions of EMPA on DOX-induced myocardial injury, histopathological examination of the rat hearts was performed.

**Figure 5:**
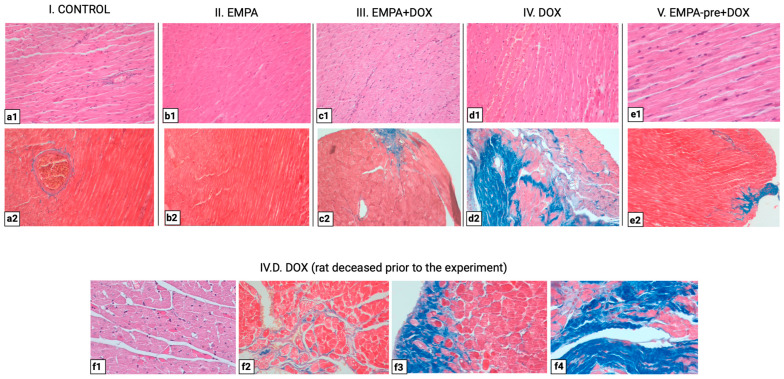
The effect of empagliflozin (EMPA) on DOX-induced cardiotoxicity in rats. Representative sections of hearts stained with hematoxylin–eosin (H&E) and Masson’s trichrome. (**a1**) Normal histological appearance of cardiomyocytes in a longitudinal section with an isolated vessel with active hyperemia and perivascular lymphocytic inflammatory infiltrate (H&E; Ob. 20×). (**a2**) Cardiac muscle fibers are highlighted in red; fibrous connective tissue in the vessel wall is stained blue, and absent in the myocardial tissue (Masson’s trichrome; Ob. 20×). (**b1**) Cardiac muscle fibers in longitudinal section, with preserved longitudinal striations representing myofibrils (H&E; Ob. 20×). (**b2**) Cardiac muscle fibers are highlighted in red; fibrous connective tissue is absent in the myocardial tissue (Masson’s trichrome; Ob. 20×). (**c1**) Cardiomyocytes with diffuse lymphocytic inflammatory infiltrate, with elongated thin cardiomyocytes (H&E; Ob. 20×). (**c2**) Diffuse interstitial fibrosis, less than in group IV (Masson’s trichrome; Ob. 20x). (**d1**) Elongated, thinned cardiomyocytes with loss of striations and perinuclear vacuolization; diffuse lymphocytic inflammatory infiltrate in the interstitium (H&E; Ob. 20×). (**d2**) Extensive interstitial fibrotic areas with focal entrapment and cardiomyocyte atrophy (Masson’s trichrome; Ob. 20×). (**e1**) Cardiomyocytes in longitudinal section with diffuse inflammatory infiltrate (H&E; Ob. 40×). (**e2**) Diffuse interstitial fibrosis, less than in group IV (Masson’s trichrome; Ob. 10×). (**f1**–**f4**) H&E staining (Ob. 40×) and Masson’s trichrome staining (Ob. 20×, Ob. 40×) of a heart from group IV, a rat that died before the experiment; loss of striations with vacuolization of cardiomyocytes, diffuse inflammatory infiltrate, and extensive areas of interstitial fibrosis causing constriction of the cardiomyocytes.

Regarding the acute reaction pattern, in the group treated with DOX (group IV), cytoplasmic vacuolization of cardiomyocytes and loss of transverse striations were observed, histological changes compatible with anthracycline cardiotoxicity, which were not observed in the groups treated concomitantly or preconditioned with EMPA (groups III and V), nor in the control group or in the EMPA monotherapy group. Regarding the inflammatory component, diffuse inflammation was observed in all DOX-exposed groups, regardless of EMPA combination, whereas it was absent in groups I and II. Thinning and elongation of cardiomyocytes were also noted, suggestive of maladaptive remodeling, predominantly observed in groups III and IV (Figure 5).

In the chronic response pattern, histological analysis revealed more pronounced myocardial fibrosis in group IV, with extensive interstitial collagen deposition, compared with groups III and V, in which rats benefited from EMPA-induced cardioprotection. In groups exposed to DOX and EMPA, fibrosis was reduced in extent and severity, suggesting attenuation of myocardial structural remodeling processes under the effect of the SGLT2 inhibitor. In addition, cardiomyocyte atrophy was characteristic of group IV (Figure 4).

Both acute and chronic histological changes have been summarized in the table below to allow for easier and comparative assessment of the cardioprotective effect of EMPA on the DOX-induced cardiotoxicity model (Table 3).

## 3. Discussion

In this experimental study, designed to evaluate the effect of EMPA on DOX-induced cardiotoxicity, EMPA demonstrated a consistent cardioprotective profile. At the ECG level, DOX exposure was associated with QTc prolongation and repolarization abnormalities, suggestive of increased arrhythmic vulnerability, while EMPA-treated groups, either concomitantly or preconditioned, showed attenuation of these abnormalities, suggesting a favorable effect on myocardial electrical stability. Troponin I levels were significantly elevated in group IV, reflecting extensive myocyte injury, whereas a more moderate increase in troponin was observed in the groups receiving EMPA in combination. Histopathologic analysis showed cardiomyocyte vacuolization and loss of striations, diffuse inflammation, diffuse cardiomyocyte atrophy, and marked fibrosis in group IV, whereas in the EMPA groups, these lesions were attenuated. Taken together, these results support the idea that EMPA may significantly reduce anthracycline cardiotoxicity, making this agent of particular interest in cardio-oncology.

Researchers have created many animal models to study DOX-induced heart damage, using different methods and doses to reflect either acute or chronic conditions seen in patients. For example, Podyacheva et al. described a protocol using Wistar rats and gave them DOX via injection six times every other day, with total doses of 10–20 mg/kg, to induce both acute and chronic cardiomyopathy [25]. Timm et al. used male Wistar rats treated with weekly intravenous doses of 2–3 mg/kg for 5–6 weeks (cumulative dose of 12–15 mg/kg), which led to early, detectable heart damage shown by imaging and tissue analysis [26]. Several studies have used a cumulative doxorubicin dose of 15 mg/kg in rat cardiotoxicity models, reporting acute cardiac damage and early heart failure. This approach is widely accepted as a standard preclinical model [3]. Accordingly, and consistent with the literature, a total cumulative dose of 15 mg/kg of DOX was selected for our study protocol.

Experimental and clinical studies indicate that SGLT2 inhibitors have cardioprotective effects against anthracycline-induced cardiotoxicity. These agents modulate key pathogenic pathways by attenuating the inflammatory response, reducing oxidative stress, and decreasing cardiomyocyte apoptosis. Additionally, SGLT2 inhibitors stimulate adaptive autophagy, inhibit ferroptosis, and modulate energy metabolism, thereby limiting cardiomyocyte loss and maladaptive ventricular remodeling [3]. In most rat models of DOX-induced cardiotoxicity, researchers use relatively high doses of SGLT2 inhibitors to make sure the medication has a clear pharmacological effect [27,28,29,30]. In our experimental study, we used a dose of 10 mg/kg/day of EMPA, which provided a relevant cardioprotective effect in models of DOX-induced cardiotoxicity without compromising animal tolerability. To facilitate translational interpretation, the corresponding human-equivalent dose (HED) was estimated using body surface area–based allometric scaling, following the approach described by Nair et al., with correction factor (Km) values of 6 for rats and 37 for adult humans. Using the formula HED (mg/kg) = animal dose (mg/kg) × (Km rat/Km human), the 10 mg/kg rat dose is moderately supratherapeutic but within the same order of magnitude as approved clinical empagliflozin doses [31,32]. In addition, our experimental design included both a group of rats treated concomitantly with EMPA and DOX (group III) and a group subjected to a preconditioning protocol in which EMPA was administered for 14 days prior to the initiation of DOX, followed by a further 14 days of combined EMPA + DOX treatment (group V).

We observed that DOX administration led to clear changes in ECG morphology. These included a longer PR interval, longer QT and QTc intervals, lower QRS complex amplitude, lower HR, and a nonspecific ST-segment pattern. In our DOX-treated rats, HR was lower than in the control group (276.8 ± 10.4 bpm vs. 301.0 ± 6.2 bpm; *p* = 0.0111), an apparently paradoxical finding compared with clinical observations of compensatory sinus tachycardia in anthracycline-induced HF in cancer patients [33]. However, recent preclinical data indicate that DOX can directly impair sinus node function and thereby induce bradycardia. A study published in 2025 on a mouse model found that DOX reduced intrinsic HR in both in vivo and ex vivo settings and was associated with mitochondrial fragmentation, nuclear abnormalities, and downregulation of key pacemaker genes (HCN4, SERCA2, RyR2) in the sinus node, ultimately leading to pacemaker dysfunction and bradycardia [34]. Consistent with other rodent DOX-cardiomyopathy models, these findings suggest that, in small animals, direct conduction system toxicity, autonomic imbalance, and systemic illness may predominate over classical compensatory mechanisms, leading to relative bradycardia or no significant change in HR [35,36]. The decreased HR observed in our DOX group may reflect DOX-induced pacemaker dysfunction, but it should not be directly extrapolated to HR responses in human patients after chemotherapy.

In a rat model, Satyam et al. studied dapagliflozin (DAPA) co-administered with DOX and found that the QT, QTc, and PR intervals were significantly decreased in DAPA-treated rats (0.9 mg/kg) compared with DOX control rats [37]. Barış et al. monitored ECG changes in rats treated with empagliflozin. The group that received only DOX showed significant prolongation of the PR, QT, and QTc intervals (*p* < 0.001), suggesting anthracycline cardiotoxicity. In contrast, the group given both DOX and EMPA had much less QT and QTc prolongation, supporting the SGLT2 inhibitors’ protective effect on ventricular repolarization. This suggests its protective effect on ventricular repolarization comes mainly from regulating ion movement and preventing problems with excitation-contraction coupling, rather than from a general metabolic effect [24]. It appears that DOX-induced cardiotoxicity involves damage to the sarcoplasmic reticulum, inhibition of SERCA (Sarcoplasmic/Endoplasmic Reticulum Calcium ATPase), and ryanodine receptor activation, which raises cytosolic Ca^2+^ levels and prolongs the QT/QTc interval [38,39]. EMPA helps restore calcium balance, lower intracellular Na^+^/Ca^2+^, and reduce abnormal Ca^2+^ release from the reticulum [40]. In our study, EMPA treatment substantially attenuated DOX-induced electrocardiographic alterations. Heart rate remained comparable to control values, QRS duration was reduced, and both PR and QTc intervals showed marked improvement. The R-wave amplitude was preserved, approaching normal values, and the T-wave amplitude remained positive.

Doxorubicin administration in rats results in a distinct, dose- and time-dependent elevation of myocardial damage indicators, which reflects myocyte injury in both acute and chronic cardiotoxicity. Doxorubicin increased the level of TnI in our findings. This is consistent with earlier research suggesting that DOX-induced oxidative stress can result in cardiac troponin release, proportional to the size and extent of cardiac tissue injury [22,30,41]. The present study demonstrates a robust cardioprotective effect of SGLT2 inhibitors, as troponin I levels were significantly lower in both the EMPA + DOX group (*p* = 0.037) and the EMPA preconditioning + DOX group (*p* = 0.010) compared with the DOX monotherapy group.

Our study found that giving Sprague-Dawley rats a total dose of 15 mg/kg DOX caused significant myelosuppression and severe pancytopenia. This matches earlier reports of strong bone marrow depression and cytopenias after similar anthracycline exposure in rodents [25]. To the best of our knowledge, no studies have shown that EMPA or other SGLT2 inhibitors modulate or ameliorate this DOX-induced myelosuppression; instead, the available literature describes a predominantly cardiac-selective protective effect, mediated through pathways related to myocardial metabolism, oxidative stress, and inflammatory signaling, without evidence for direct protection of proliferating hematopoietic cells.

Studying histologic changes in DOX-induced cardiotoxicity is important because it shows the structural damage behind ventricular dysfunction, ECG changes, and serum markers like troponin. Microscopy helps identify the stages of damage, from vacuolization and edema to cardiomyocyte necrosis and fibrosis, and distinguishes between acute injury and chronic remodeling [18,22,24,37,42]. When these findings are combined with experiments using SGLT2 inhibitors, researchers can see not only the functional effects but also whether these drugs reduce cardiomyocyte loss, inflammation, and cardiac fibrosis, thereby changing the course of cardiotoxicity [43]. In our experimental model, histological analysis revealed cytoplasmic vacuolization of cardiomyocytes, loss of cross-striations, and extensive infiltration of inflammatory cells in the DOX group. These pathological changes were largely absent in the control and empagliflozin-only groups. In both the EMPA + DOX and EMPA-pre + DOX groups, the severity of these injuries was reduced, with improved preservation of cardiomyocyte structure and decreased inflammation. Regarding chronic changes, DOX administration led to marked fibrosis and cardiomyocyte atrophy in the DOX group. In contrast, animals treated with empagliflozin exhibited significantly less collagen deposition and no evident cardiomyocyte atrophy, indicating attenuation of adverse structural remodeling.

Altogether, our data provide an integrated view of DOX cardiotoxicity and EMPA protective effects by linking serial ECG changes, cardiac troponin I elevations, and both acute and chronic histopathological findings within the same in vivo model, rather than examining these areas separately. Including a preconditioning group, in which EMPA is initiated before DOX treatment and continued during DOX treatment, helps address a timing question that has not been fully explored in prior EMPA/DOX studies. In our experimental setting, EMPA preconditioning did not deliver additional statistical cardioprotection compared with its concomitant initiation alongside DOX. These findings indicate that initiating EMPA at the start of anthracycline therapy may be a more pragmatic approach than implementing a separate pretreatment phase.

A limitation of this study is that echocardiography was not performed. Ventricular function was assessed based on ECG, cardiac enzymes, and histopathological changes. Future studies from our group will complement these data with real-time evaluation of cardiac kinetics during open-chest experiments under intubation and mechanical ventilation, using a high-resolution 3D camera and machine learning techniques.

We should specify another limitation of the study: the small final sample sizes in the DOX (*n* = 4) and EMPA + DOX (*n* = 5) groups due to treatment-related mortality, which may reduce statistical power; therefore, the results in these groups should be confirmed in larger studies.

Finally, we did not perform dedicated mechanistic assays (oxidative stress, inflammatory signaling, or mitochondrial dysfunction), so our data should be interpreted as a detailed in vivo phenotypic characterization that complements existing work that elucidates the molecular pathways of DOX cardiotoxicity and EMPA-mediated cardioprotection.

## 4. Materials and Methods

### 4.1. Animals

The study involved 30 adult male Sprague-Dawley rats, each weighing 400–450 g, obtained from the Cantacuzino National Research Institute, Bucharest, Romania. The animals were kept under controlled conditions: 12 h of light and 12 h of darkness, ambient temperature (22 ± 1 °C), and free access to water and standard laboratory food. The experiment followed ethical principles and international standards for animal protection. The protocol was approved by the Institutional Ethics Committee (No. 40/23.07.2025) of Victor Babeș University of Medicine and Pharmacy, Timisoara, Romania, in accordance with institutional guidelines for the care and use of laboratory animals. No human data or tissue were included in this investigation.

### 4.2. Dox-Induced Cardiotoxicity in Sprague-Dawley Rats

**Figure 6:**
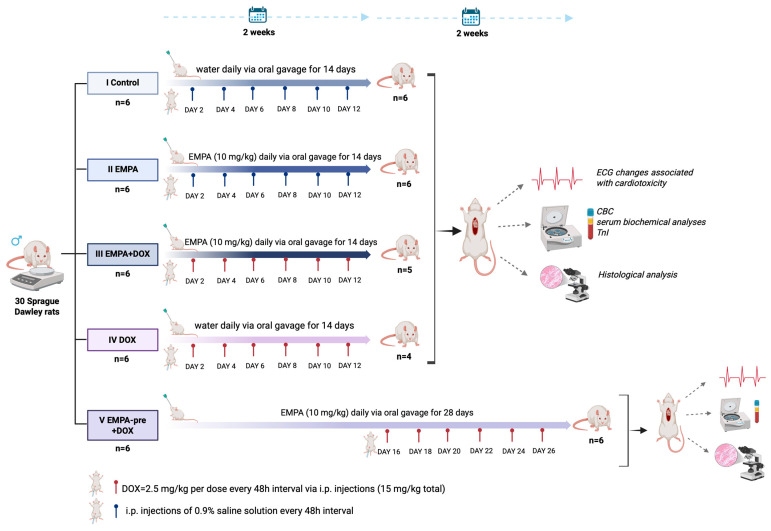
Study design. CBC = complete blood count; TnI = troponin I; DOX = doxorubicin; ECG = electrocardiogram; EMPA = empagliflozin; i.p. = intraperitoneal. Created in BioRender (https://BioRender.com/jic43md) (accessed on 18 March 2026).

The 30 animals were randomly assigned to 5 experimental groups, with six rats per group. The protocol employed a cardiotoxicity model with varying treatment regimens and observation periods, as detailed below (Figure 6):

Group I (Control): Animals received daily administration of water via oral gavage for 14 consecutive days, with intraperitoneal injections (i.p.) of 0.9% saline solution at 48 h intervals (days 2, 4, 6, 8, 10, and 12).

Group II (Empagliflozin): Animals received Empagliflozin (Boehringer Ingelheim, Ingelheim am Rhein, Germany) (10 mg/kg) daily via oral gavage for 14 days, simultaneously with i.p. injections of saline at 48 h intervals.

Group III (Empagliflozin + Doxorubicin): Animals received EMPA (10 mg/kg) daily via oral gavage throughout the 14-day period, combined with i.p. DOX administration (2.5 mg/kg) at 48 h intervals for a total of six doses, achieving a cumulative DOX dose of 15 mg/kg.

Group IV (Doxorubicin): Animals received water daily by oral gavage for 14 days and i.p. DOX injections (2.5 mg/kg) at 48 h intervals, resulting in a cumulative dose of 15 mg/kg.

For groups I to IV, experiments concluded on day 14.

Group V (Empagliflozin Preconditioning + Doxorubicin): Animals underwent a preconditioning phase with daily EMPA administration (10 mg/kg) via oral gavage for 14 days, followed by the EMPA/DOX protocol over the subsequent 14 days. This group allowed evaluation of cardioprotective effects of prior EMPA exposure. Total experimental duration: 28 days.

Doxorubicin hydrochloride (Doxorubicin Accord, 2 mg/mL; Accord Healthcare Ltd., North Harrow, Middlesex, UK), a clear red solution with a pH of 2.5–3.5, was diluted ex tempore in sterile 0.9% sodium chloride (normal saline; 0.9% NaCl; B. Braun, Melsungen, Germany) and administered intraperitoneally at a dose of 2.5 mg/kg in a volume of 1 mL/kg using an insulin syringe, without additional pH adjustment.

### 4.3. Collection of Samples

All rats were weighed on day 1 to record their baseline weight. General anesthesia was induced and maintained with isoflurane (AbbVie Deutschland GmbH & Co. KG, Wiesbaden, Germany) in oxygen at 5% for induction and 2–2.5% for maintenance, to maintain a stable anesthetic plane during surgical procedures.

**Figure 7:**
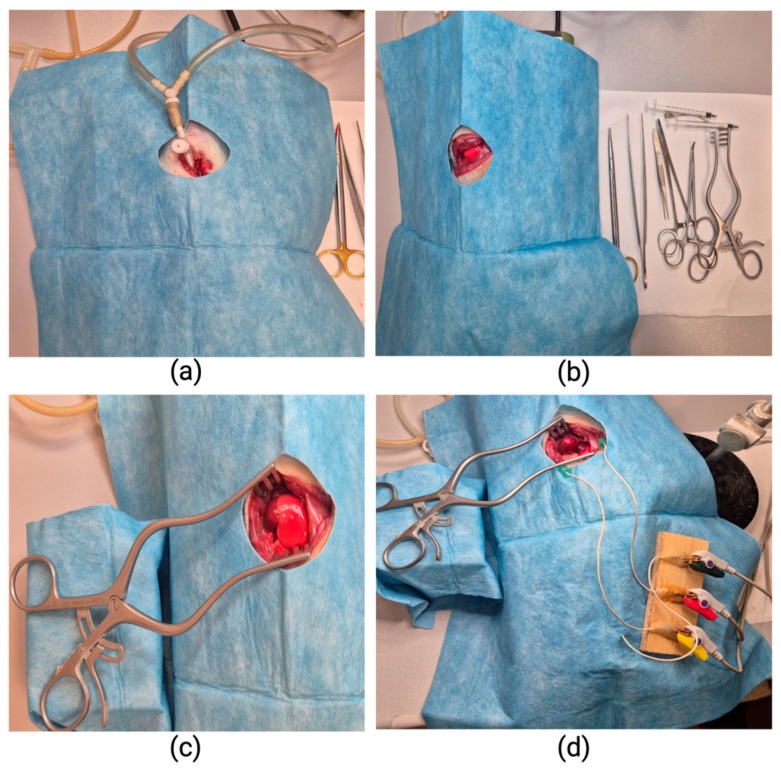
Anesthetic–surgical protocol: (**a**) The rat is intubated via tracheostomy. (**b**) Lateral thoracotomy allows exposure of the heart. (**c**) A retractor is positioned to improve visualization of the heart, and attempts are made to isolate it from the lung to avoid interference with subsequent video recording (for further research). (**d**) Subcutaneous electrodes are attached for continuous monitoring of cardiac electrical activity.

After induction of anesthesia, a tracheostomy was performed, and the trachea was cannulated with a 14-G peripheral venous catheter, which allowed for assisted mechanical ventilation. Subsequently, a left lateral thoracotomy was performed in the V intercostal space, with controlled opening of the chest cavity. The pericardium was identified and sectioned, thereby providing direct exposure of the heart, particularly the left ventricle (Figure 7).

To monitor cardiac electrical activity, electrodes for the Einthoven DII lead were placed, and the electrocardiogram was continuously recorded using a CONTEC Monitor CMS6000 (Contec Medical Systems Co., Ltd., Qinhuangdao, Hebei, China). The operating table was maintained at 36 °C to prevent hypothermia and maintain stable physiological conditions. After thoracotomy, hemodynamic stabilization was achieved, and electrocardiographic signals were acquired for a standardized duration of 5 min per animal. After that, the inferior vena cava (IVC) was surgically accessed, and venous blood was collected for complete blood count (CBC) and biochemical analyses.

After completion of the collection, potassium chloride (Sigma-Aldrich, St. Louis, MO, USA) was administered intravenously at a dose of 100 mg/kg for euthanasia of the animals. Immediately after cardiac arrest, the heart was removed and processed for further analysis.

### 4.4. Electrocardiographic Data

The ECG recordings were taken from lead DII using needle electrodes. Data Acquisition and Network Interception Electrocardiographic (ECG) signals were acquired via direct digital interception of the device’s Ethernet output to ensure maximum signal fidelity and prevent the data loss associated with traditional analog-to-digital conversion. Raw network traffic was monitored and captured with Wireshark (v4.6.3), yielding high-resolution packet capture (.pcap) files. These files contained the encapsulated binary stream of the heart’s electrical activity as transmitted by the acquisition hardware. The raw packet payloads were extracted and translated into an ASCII-based coordinate format (.asc), representing voltage over time. This raw signal was processed using the open-source ecg_processing framework (WFDB Python package, version 4.3.0, GitHub), which was used to filter baseline noise, detect R peaks, and isolate individual cardiac cycles (P-QRS-T complexes) from the continuous data stream.

The ECGs were analyzed by cardiologists from our research team, experienced in interpreting electrocardiographic changes associated with cardiotoxicity. The evaluation included both quantitative and qualitative analysis. Heart rate, PR, QT intervals, and QRS complex duration were measured. The corrected QT interval (QTc) was calculated using the Bazett formula. Qualitatively, ST-segment changes, T wave alterations, and any other features suggestive of doxorubicin-induced myocardial damage were monitored.

### 4.5. Blood Sample Analyses

Venous blood samples were collected from the IVC of each rat at the end of the protocol for complete blood count (CBC), serum biochemical analyses, and cardiac troponin dosage, in accordance with preanalytical stability recommendations for the biomarkers analyzed. Blood for CBC was collected in K2-EDTA tubes and analyzed promptly. Serum and plasma were obtained by centrifugation of the blood at 3000 rpm for 15 min at room temperature (Centrifuge 5810, Eppendorf AG, Hamburg, Germany) and then stored at −20 °C for further biochemical analysis.

### 4.6. Histological Assessment

At the end of the experiments, the rats were sacrificed, and the heart tissue was collected for histological analysis. The hearts were sectioned from the base to the apex to obtain representative left ventricular tissue, which was fixed in 4% buffered formaldehyde (Roti-Histofix, Carl Roth GmbH, Karlsruhe, Germany) and embedded in paraffin, according to standard protocols. Hematoxylin–eosin (H&E) staining (Sigma-Aldrich, St. Louis, MO, USA) was used to evaluate the overall architecture of the myocardium and to identify changes characteristic of anthracycline cardiotoxicity in the acute phase. To assess chronic changes, particularly fibrotic remodeling in the context of doxorubicin exposure, Masson’s trichrome staining (Bio-Optica, Milano, Italy) was used, which allows differentiation between myocardial fibers (cardiac cytoplasm stains red and nuclei stain black) and fibrotic tissue (collagen fibers stain blue). An experienced pathologist, unaware of which treatment each group received, examined myocardial sections from all groups. Lesions were divided into acute and chronic reaction patterns. Acute changes included cardiomyocyte vacuolization and loss of transverse striations, inflammatory infiltration (both diffuse and spotty), and thinned, elongated cardiomyocytes. Chronic changes included fibrosis, which was graded by the amount of collagen seen at high magnification (MF, 40×) and marked as “>” if it involved more than one microscopic field or “<” if it was limited to less than one field, as well as cardiomyocyte atrophy. The severity of these lesions was graded semi-quantitatively based on how much of the tissue was affected: “−” for normal (no lesion), “+” for mild, “++” for moderate, and “+++” for severe.

### 4.7. Statistical Analysis

All data were analyzed by comparing the five groups. Continuous variables are presented as mean ± standard deviation (SD). First, for each ECG and biochemical parameter, we used a one-way ANOVA to check for overall differences between groups. The weight change was calculated by dividing the final weight by the baseline weight. Then, we focused on three planned comparisons: group IV versus group I (to demonstrate the cardiotoxic effect of doxorubicin), group IV versus group III (to evaluate the effect of concomitant empagliflozin administration), group IV versus group V (to evaluate the effect of prior empagliflozin administration), and group III versus group V (to compare the two EMPA regimens). For these comparisons, independent-samples *t*-tests were used, with a significance threshold of *p* < 0.05; *p*-values < 0.001 were considered highly significant. The difference between baseline and final weight was determined using repeated-measures ANOVA, with treatment as a predictor. The comparison of these weight differences was further evaluated for each treatment using a paired-samples *t*-test. Statistical analyses were performed using IBM SPSS Statistics, version 24.0 (SPSS Inc., Chicago, IL, USA). The graphs were created using GraphPad Prism, version 9.3.0.

## 5. Conclusions

The findings indicate that empagliflozin (10 mg/kg) offers significant cardioprotection against doxorubicin-induced cardiotoxicity (15 mg/kg cumulative dose) in Sprague-Dawley rats. This is demonstrated by improvement in ECG parameters, reduction in troponin levels, and attenuation of myocardial histopathologic lesions. Within this experimental framework, our study demonstrates: (i) an integrated, multi-level characterization of cardioprotection in a non-diabetic doxorubicin rat model; (ii) a direct head-to-head comparison of two clinically relevant timing strategies of empagliflozin administration that both use a high cumulative doxorubicin dose with documented pancytopenia; and (iii) a systematic demonstration that empagliflozin improves electrical, biochemical, and structural parameters of cardiac injury without mitigating doxorubicin-induced myelosuppression, supporting a cardioprotective effect that does not compromise systemic anticancer efficacy. In conclusion, it provides a clinically relevant therapeutic framework that may advise future cardio-oncology trials and treatment algorithms for patients exposed to anthracyclines.

## Figures and Tables

**Table 1:** Electrocardiographic findings.

	Group I Control (*n* = 6)	Group II EMPA (*n* = 6)	Group III EMPA + DOX (*n* = 5)	Group IV DOX (*n* = 4)	Group V EMPA-Pre + DOX (*n* = 6)	IV vs. I	IV vs. III	IV vs. V	III vs. V
HR (bpm) (mean ± SD)	301.0 ± 6.2	296.3 ± 6.5	297.0 ± 2.0	276.8 ± 10.4	288.3 ± 12.9	0.0111	0.0283	0.1611	0.1611
QRS segment (ms) (mean ± SD)	19.98 ± 1.10	22.76 ± 2.27	22.48 ± 1.75	29.65 ± 1.80	22.37 ± 1.05	0.0004	0.0007	0.0013	0.9057
PR interval (ms) (mean ± SD)	42.15 ± 1.68	46.07 ± 3.10	45.73 ± 2.44	57.80 ± 2.91	45.50 ± 1.30	0.0004	0.0006	0.0017	0.8561
QT interval (ms) (mean ± SD)	74.98 ± 2.50	81.54 ± 3.07	87.44 ± 4.22	125.10 ± 7.89	85.84 ± 3.39	0.0006	0.0007	0.0010	0.5143
QTc interval (ms) (mean ± SD)	167.9 ± 4.7	181.2 ± 6.2	194.5 ± 9.3	268.7 ± 19.4	188.1 ± 6.5	0.0014	0.0020	0.0024	0.2357
R amplitude (mV) (mean ± SD)	0.87 ± 0.06	0.81 ± 0.03	0.87 ± 0.03	0.57 ± 0.03	0.82 ± 0.06	0.0001	0.00003	0.0006	0.1132
T amplitude (mV) (mean ± SD)	0.34 ± 0.01	0.33 ± 0.03	0.34 ± 0.03	−0.19 ± 0.03	0.34 ± 0.03	0.0013	0.0002	0.0002	>0.9999

bpm = beats per minute; HR = heart rate; ms = milliseconds; mV = millivolt; *n* = number of subjects; SD = standard deviation; Group I (Control); Group II EMPA (Empagliflozin); Group III EMPA + DOX (Empagliflozin + Doxorubicin); Group IV DOX (Doxorubicin); Group V EMPA-pre + DOX (Empagliflozin Preconditioning + Doxorubicin); QTc was calculated with the Bazett formula (QTc = QT/$\sqrt{RR}$).

**Table 2:** Blood sample findings.

	Group I Control (*n* = 6)	Group II EMPA (*n* = 6)	Group III EMPA + DOX (*n* = 5)	Group IV DOX (*n* = 4)	Group V EMPA-Pre + DOX (*n* = 6)	IV vs. I	IV vs. III	IV vs. V	III vs. V
WBC (10^9^/L) (mean ± SD)	6.21 ± 2.27	7.59 ± 1.80	0.64 ± 0.36	0.57 ± 0.23	0.76 ± 0.30	0.002	0.734	0.292	0.5697
RBC (10^12^/L) (mean ± SD)	8.35 ± 0.63	7.84 ± 1.10	5.13 ± 0.77	4.86 ± 0.47	5.17 ± 0.69	<0.001	0.539	0.423	0.9305
HGB (g/dL) (mean ± SD)	14.40 ± 0.86	13.80 ± 1.62	8.68 ± 1.10	8.20 ± 0.62	8.80 ± 1.25	<0.001	0.439	0.346	0.8693
PLT (10^9^/L) (mean ± SD)	961.0 ± 53.6	920.3 ± 199.3	37.8 ± 20.6	35.0 ± 28.0	42.0 ± 20.0	<0.001	0.873	0.684	0.7412
AST (U/L) (mean ± SD)	93.58 ± 9.22	85.95 ± 8.92	138.37 ± 18.3	147.7 ± 17.6	146.1 ± 29.1	0.004	0.464	0.916	0.6058
ALT (U/L) (mean ± SD)	57.73 ± 16.9	36.59 ± 5.76	31.71 ± 2.21	28.34 ± 3.91	35.61 ± 10.6	0.007	0.191	0.171	0.4162
LDH (U/L) (mean ± SD)	359.1 ± 55.5	424.5 ± 116.5	131.7 ± 23.0	133.5 ± 17.6	128.1 ± 26.1	<0.001	0.898	0.706	0.8134
TP (g/dL) (mean ± SD)	5.96 ± 0.85	5.20 ± 0.09	3.98 ± 0.60	4.39 ± 0.46	4.75 ± 0.34	0.006	0.284	0.236	0.0431
TnI (ng/mL) (mean ± SD)	0.09 ± 0.02	0.07 ± 0.02	0.14 ± 0.04	0.20 ± 0.03	0.13 ± 0.02	0.002	0.037	0.010	0.6303

ALT = alanine aminotransferase; AST = aspartate aminotransferase; HGB = hemoglobin; LDH = lactate dehydrogenase; PLT = platelets; SD = standard deviation; RBC = red blood cells; TnI = troponin I; TP = total proteins; WBC = white blood cells.

**Table 3:** Pattern of acute and chronic histopathological reactions in rat hearts.

	Acute Reaction Pattern			Chronic Reaction Pattern		Other Changes
	Cardiomyocyte vacuolization/loss of striations	Diffuse inflammation (d)/spots of inflammation (s)	Thinned, elongated cardiomyocytes	Fibrosis **>**(more than one MF 40×) **<**(less than one MF 40×)	Cardiomyocyte atrophy	
I. CONTROL	−	−	−	−	−	
II. EMPA	−	−	−	−	−	
III.EMPA + DOX	−	(d) +	+	<	−	Hyperemia
IV. DOX	++	(d) ++	++	>	++	
V. EMPA-pre +DOX	−	(d) +	−	<	−	Pericardial lipomatosis

Acute reaction pattern includes cardiomyocyte vacuolization/loss of striations, diffuse inflammation (d)/spots of inflammation (s), and thinned, elongated cardiomyocytes; chronic reaction pattern includes fibrosis (>, more than one MF at 40×; <, less than one MF at 40×) and cardiomyocyte atrophy. The severity of each lesion was graded as − (normal), + (mild), ++ (moderate).

## Data Availability

The original contributions presented in the study are included in the article. Further inquiries can be directed to the corresponding authors.

## Abbreviations

The following abbreviations are used in this manuscript:

ALT	Alanine aminotransferase
AMPK	Adenosine monophosphate-activated protein kinase
AST	Aspartate aminotransferase
Bpm	Beats per minute
CBC	Complete blood count
CTRCD	Cancer therapy-related cardiac dysfunction
DOX	Doxorubicin
ECG	Electrocardiography
EMPA	Empagliflozin
GDMT	Guided directed medical therapy
H&E	Hematoxylin–eosin
HED	Human equivalent dose
HF	Heart Failure
HFpEF	Heart Failure with preserved ejection fraction
HFrEF	Heart Failure with reduced ejection fraction
HGB	Hemoglobin
HR	Heart rate
IVC	Inferior vena cava
i.p.	intraperitoneal
LDH	Lactate dehydrogenase
ms	Milliseconds
mV	Millivolt
PLT	Platelets
QTc	Corrected QT
RBC	Red blood cells
SD	Standard deviation
SGLT2	Sodium-glucose cotransporter 2
T2DM	Type 2 diabetes mellitus
TnI	Troponin I
TP	Total proteins
WBC	White blood cells
